# Signaling Role of Prokineticin 2 on the Estrous Cycle of Female Mice

**DOI:** 10.1371/journal.pone.0090860

**Published:** 2014-03-14

**Authors:** Ling Xiao, Chengkang Zhang, Xiaohan Li, Shiaoching Gong, Renming Hu, Ravikumar Balasubramanian, William F. Crowley W. Jr., Michael H. Hastings, Qun-Yong Zhou

**Affiliations:** 1 Department of Pharmacology, University of California, Irvine, California, United States of America; 2 Department of Endocrinology, Jinshan Hospital affiliated to Fudan University, Shanghai, China; 3 GENSAT Project, The Rockefeller University, New York, New York, United States of America; 4 Institute of Endocrinology and Diabetology, Huashan Hospital affiliated to Fudan University, Shanghai, China; 5 Harvard Reproductive Endocrine Sciences Center & The Reproductive Endocrine Unit, Massachusetts General Hospital, Boston, Massachusetts, United States of America; 6 Division of Neurobiology, Medical Research Council Laboratory of Molecular Biology, Cambridge, United Kingdom; University of Texas Southwestern Medical Center, United States of America

## Abstract

The possible signaling role of prokineticin 2 (PK2) and its receptor, prokineticin receptor 2 (PKR2), on female reproduction was investigated. First, the expression of PKR2 and its co-localization with estrogen receptor (ERα) in the hypothalamus was examined. Sexually dimorphic expression of PKR2 in the preoptic area of the hypothalamus was observed. Compared to the male mice, there was more widespread PKR2 expression in the preoptic area of the hypothalamus in the female mice. The likely co-expression of PKR2 and ERα in the preoptic area of the hypothalamus was observed. The estrous cycles in female PK2-null, and PKR2-null heterozygous mice, as well as in PK2-null and PKR2-null compound heterozygous mice were examined. Loss of one copy of PK2 or PKR2 gene caused elongated and irregular estrous cycle in the female mice. The alterations in the estrous cycle were more pronounced in PK2-null and PKR2-null compound heterozygous mice. Consistent with these observations, administration of a small molecule PK2 receptor antagonist led to temporary blocking of estrous cycle at the proestrous phase in female mice. The administration of PKR2 antagonist was found to blunt the circulating LH levels. Taken together, these studies indicate PK2 signaling is required for the maintenance of normal female estrous cycles.

## Introduction

The estrous cycle is an important characteristic of the mammalian female reproductive system. It is known that the estrous cycle in female rodents is under the control of both the circadian clock and hormonal changes [1]. A gonadotrophin-releasing hormone (GnRH) surge released from the hypothalamus before estrus is required to induce the pituitary to release luteinizing hormone (LH) and follicle stimulating hormone (FSH), which then influence estrous cycling. Coordinated GnRH release on the afternoon of proestrus requires both a daily timing signal originating from the suprachiasmatic nucleus (SCN) and permissive levels of estrogen and progesterone[2]. During the follicular phase of the ovarian cycle, estrogen gradually increases. As the dominant follicle continues to grow and develop, there is a change from relative inhibition of GnRH secretion to a positive concerted stimulation, resulting in the surge release of LH from the pituitary and subsequent ovulation of mature oocytes. In mice and rats, the LH surge commences in the afternoon with ovulation occurring in the middle of the night [1]. SCN lesion results in the loss of the gating response to elevated estrogen in rats[3]. Consistent with the proposed gating effect of SCN clock, prolonged and irregular estrous cycles has been reported in Bmal1-null mice that are deficient in circadian rhythms[4], [5].

The loss of circadian LH secretion and reproductive cycles abolished by the SCN lesion are not restored by SCN transplants [6], indicating specific neural efferents from the SCN might carry the output signal for the timing of the GnRH release. At the molecular level, several secretory molecules (vasopressin, vasoactive intestinal polypeptide, PK2, and cardiotrophin) have been shown to function as signaling molecules that convey SCN timing to the generation of overt circadian rhythms such as locomotor and sleep/wake cycles, body temperature and metabolic rhythms [6]–[11]. Of these, vasopressin and vasoactive intestinal polypeptide have been implicated as the SCN output signals that link the SCN clock to the normal expression of female estrous cycle [1], [2], [7], [8].

A series of studies, including gene disruption, have established PK2 as a SCN output molecule that is required for normal expression of circadian rhythms [10], [12], [18]. The disruption of PK2 and PKR2 genes has revealed an unexpected developmental role of PK2/PKR2 signaling in sexual maturation [13], [14]. As PK2 signaling via PKR2 is essential for the morphogenesis of the olfactory bulb (OB), and the OB is part of the migratory path of GnRH neurons from the nasal cavity to the hypothalamus, migration of GnRH neurons to their final hypothalamic destiny is blocked in the absence of PK2/PKR2 signaling. Therefore, PK2-null and PKR2-null mice exhibit hypogonadotropic hypogonadism and hypoplasia of reproductive organs in both male and female mice. The hypogonadism of PK2-null and PKR2-null mutations has also been verified by diverse studies of human hypogonadism carried out by several laboratories [13], [14], [15], [16]. However, the expression of PKR2 in the preoptic area of the hypothalamus of adult mice suggests that PK2 signaling may have a direct signaling role in the control of the reproductive axis [10], in addition to its indirect developmental effect. This notion was also supported by the observations that human subjects with heterozygous mutations in PK2 or PKR2 presented with complete isolated GnRH deficiency[17]. These studies have implicated a possible signaling role of PK2 in the normal control of reproduction, in addition to its role in the development of GnRH neurons.

The developmental defect of GnRH neurons in PK2-null as well as PKR2-null mice has precluded the use of such completely null mutant mice to examine the signaling role of PK2 at adult-stage physiology. We thus examined the estrous cycle alterations in heterozygous PK2-null and PKR2-null mice, as well in PK2-null and PKR2-null compound heterozygous mice. We also took advantage of a newly developed small molecule PKR2 antagonist to explore the PK2 signaling roles in the reproductive axis of female mice.

## Materials and Methods

### Mice and housing conditions

Mice were maintained in regular 12 h light (ZT0-ZT12): 12 h dark cyles (ZT12-ZT24) with light on at 6 AM. The light intensity of mouse housing cages was ∼250 lux, with room temperature set at 21°C. Mice have free access to standard food chow (fat content 5–10% of energy). The PK2-null and PKR2-null mice were backcrossed to a C57Bl6 background for at least seven generations, as described previously [12], [18]. PKR2-GFP transgenic mice were generated in mouse strain FVB/N as described [19]. Genotyping of mice was performed by PCR analysis of DNA samples from tail biopsies. The control mice of all experiments were littermates. Female and male mice for the study were separately housed, and were 3–5 months of age. For PKR2 expression studies, male and female PKR2-GFP transgenic mice were sacrificed at 10 AM (ZT4). Female PKR2-GFP transgenic mice were sacrificed when they were in diestrous stage. All studies were approved by Institutional Animal Care and Use Committee of University of California,Irvine. Every possible effort has been undertaken to mitigate the animal suffering.

### Cytologic evaluation of the estrous cycles

Stages of the estrous cycle were determined by cytologic evaluation of vaginal smears. Briefly, sterile saline buffer was gently flushed into the vagina using soft plastic pipettes between 10 AM (ZT4) and 12 noon (ZT6) daily. The lavages were smeared on glass slides and examined microscopically to evaluate the cytologic features. The stages of the estrous cycle were determined based on the presence or absence of leukocytes, cornified epithelial, and nucleated epithelial cells, as described by Byers S.L.[20]. Regular estrous cycles appeared as constant P-E-M-D records, and the average cycle length was 4–5 days. Irregular estrous cycles presented as interrupted cycles and could stay on any of the four stages for more than two days. When the cycle stayed on one stage for more than four or five days without change, this was recorded as non-cycling or missing estrous cyclicity. Daily observations ensured at least three consecutive cycles in each animal.

### Administration of PKR2 antagonist

To observe the effect of PK2 receptor antagonist on the estrous cycle, PK2 receptor antagonist (3Cl-MPL) or vehicle (sunflower oil, Sigma, with injection volume of 100 microliter) were delivered subcutaneously at the dose of 10 mg/kg at 10 AM (ZT4) on the day of the proestrous. The PK2 receptor antagonist, 3Cl-MPL, was synthesized as described [21]. Antagonist potency was measured by an aequorin-based Ca2+ luminescent assay in CHO cells (Chinese Hamster Ovary) that stably expressed the photoprotein aequorin and PKR2, as described previously [22].

### Histological studies

Expression studies of ERα (estrogen receptor α) and PKR2 was carried out as described [15]. Briefly, mice were perfused intracardially with 50 ml 1× PBS (2.7 mM KCl; 1.8 mM KH2PO4; 10.1 mM Na2HPO4; 137 mM NaCl) followed by 50 ml 4% paraformaldehyde in 1× PBS. Brains were then post-fixed at 4°C for 24 hours in the same fixative, cryoprotected in 30% sucrose in 1× PBS for 24 to 48 hours. 40 μm coronal sections were first blocked in PBS containing 0.2% Triton X-100 (PBST) plus 10% horse serum, then incubated with rabbit anti-ERα (1∶150; HC-20, Santa Cruz Biotechnology) in PBST containing 3% horse serum at 4°C overnight. Donkey anti-rabbit IgG (1∶200, Jackson ImmunoResearch, West Grove, PA) was added after primary antibody incubation. Immunofluorescence for PKR2-GFP-expressing neurons was performed similarly except that the first antibody was chicken anti-GFP (1∶1000, Invitrogen, Carlsbad, CA) and secondary antibody was goat-anti-chicken IgG (1∶300, Jackson ImmunoResearch). Sections were counter-stained with DAPI (Vector Laboratories) and viewed with a Zeiss (Oberkochen, Germany) fluorescence microscope. Immunofluorescence for GnRH expressing neurons are the same as for ERα expressing neurons described above, except the first antibody was rabbit anti-GnRH(1∶5,000; Chemicon, Temecula, CA) instead.

### Hormonal assay

Animals receiving one subcutaneous injection of 10 mg/kg PKR antagonist (3-Cl-MPL) or vehicle (sunflower oil) at 10 AM (ZT4) on the day of proestrous were sacrificed at light-off (ZT12) and blood was collected from the trunk. LH levels were measured by RIA, which was performed by the Ligand Assay Core of the Specialized Cooperative Center for Research in Reproduction at the University of Virginia (Charlottesville, VA). The linear range of the LH assay was 0.04–37.4 ng/ml, and the intra-assay coefficient of variation was 7%.

### Cell counting

Only cells with positive signals stronger than the background were tallied for quantification. Background signals were defined by random sampling in the no staining areas of sections as well as the surrounding areas. Cell counts were performed by counting all cells within the boundaries of the preoptic area in each section as described [23].

### Statistical analyses

**Figure 1:**
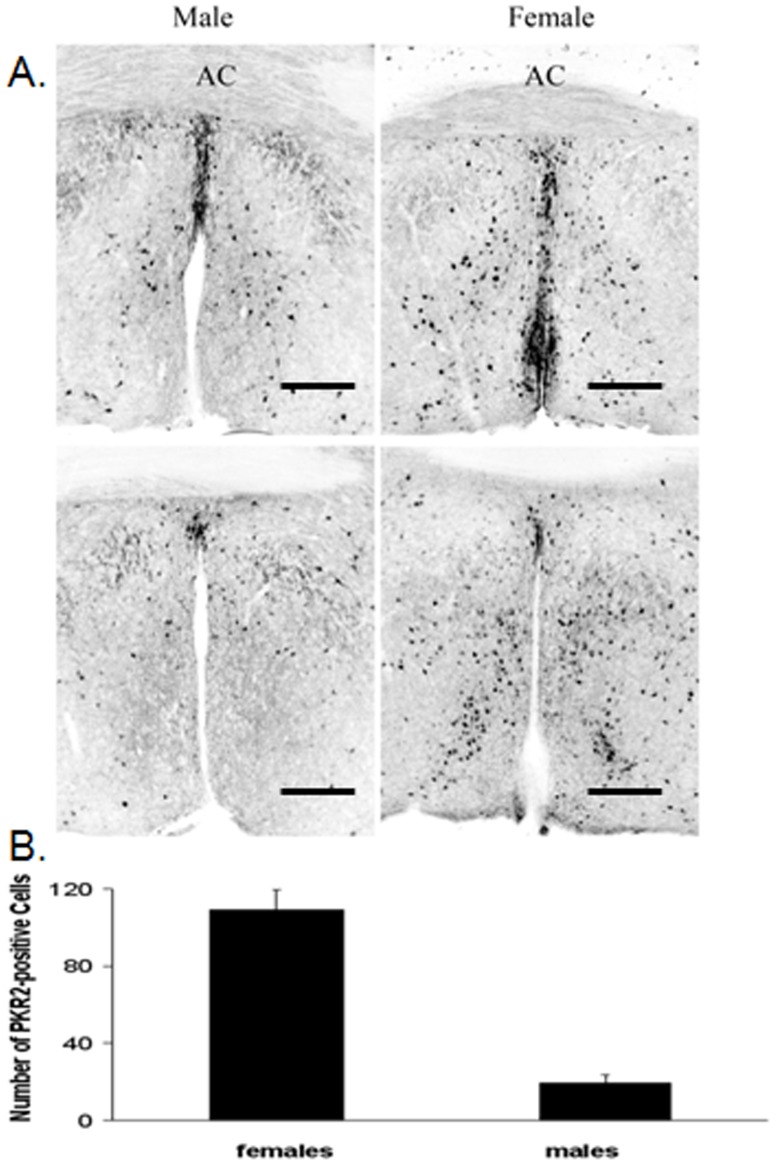
The sexually dimorphic expression of PKR2 in the preoptic area of hypothalamus. The PKR2 expression was observed with PKR2-GFP transgenic mice. Panel A shows the sexually dimorphic expression of PKR2 was apparent (scale bars:100 μm, AC: Anterior Commissure). Panel B shows Comparison of the number of PKR2-positive cells in females and males in the region of AVPV.

**Figure 2:**
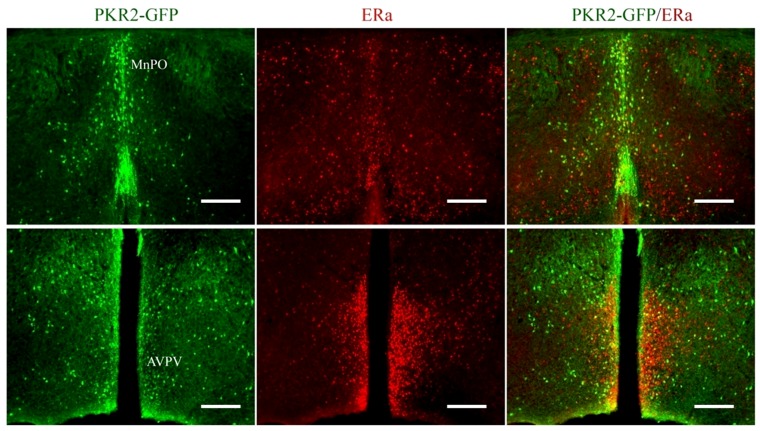
PKR2 antagonist reduced plasma LH levels (Mean ± S.E.). The LH levels in the PKR2 antagonist (10 mg/kg 3Cl-MPL) group were significantly reduced compared to the vehicle treatment (n = 6 for each group, p<0.01.

**Figure 3:**
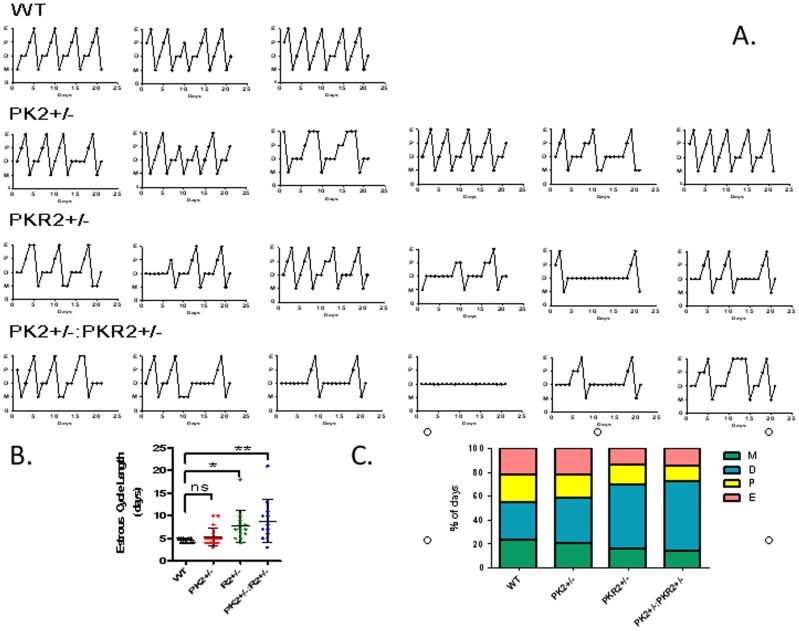
Abnormality of estrous cycle in PK2, PKR2 single and compound heterozygotic female mice. Three female mice in wild-type group, six female mice in each three mutant groups were studied. Panel A shows that three mutant groups exhibited irregular and longer estrous cycles compared to the wild-type control. PKR2 heterozygotes and the compound heterozygous female mice exhibited more pronounced abnormality. Panel B shows the quantification of estrous cycle lengths (Mean ± S.E.), * p<0.05 ** p<0.01. Panel C shows the percentage of days at each stage in 20 days of observance. D: diestrus, M: metestrus, E: estrus, P: proestrus.

**Figure 4:**
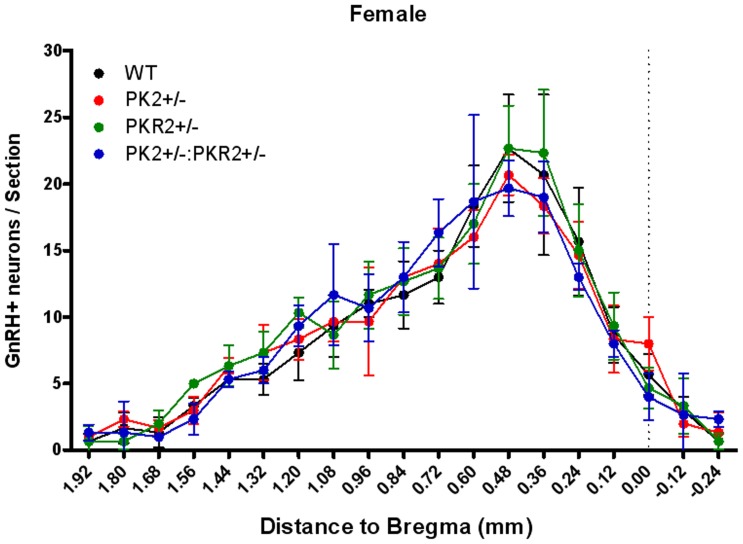
Cell counts of GnRH neurons in WT, PK2, PKR2 single and compound heterozygotic female mice(Mean ± S.E.). Two-way ANOVA with Bonferroni post-test showed no significant difference among all four groups.

Results were expressed as the mean ± SE. All statistical analyses were performed using Graphpad Prism 5. Differences between groups were examined for statistical significance by using one-way ANOVA followed by Dunnett's test (Fig.1 an Fig.2), or a two-way ANOVA for groups comparison (Fig.3 and Fig.4). P<0.05 was considered statistically significant.

## Results

**Figure 5:**
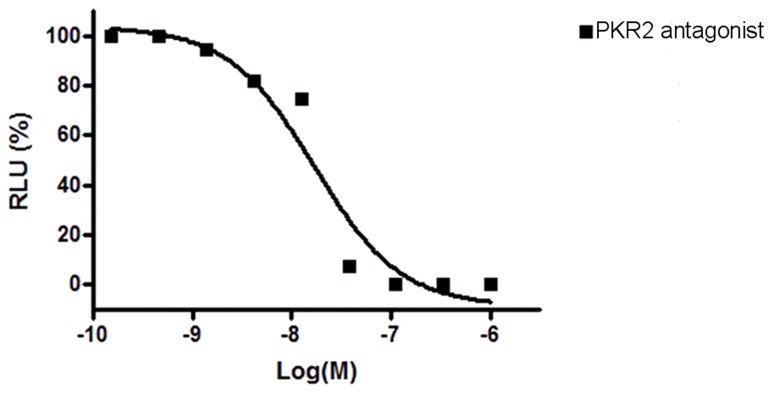
The coexpression of PKR2 and ERα in the preoptic area. PKR2 and ERα were detected by immunofluorescence staining. PKR2-GFP expression was shown in green and ERα expression was shown in red. Yellow or orange color in the MnPO and AVPV regions indicates likely co-expression of PKR2 and ERα (scale bars:100 μm).

We have previously shown that PKR2 is expressed in the preoptic area [24]. To further demonstrate PK2 signaling may regulate female reproduction, we carried out the detailed analysis of PKR2 expression in the preoptic area in both males and females. To facilitate the expression studies, we also used a line of transgenic mice with the reporter gene GFP under the control of PKR2 promoter [19]. As shown in Fig. 1A, there clearly existed sexually dimorphic expression of PKR2 in the preoptic area. Compared to male mice, females have much more widespread PKR2-expressing neurons in the preoptic area. Quantitative analysis indicated there was about six times more PKR2-positive cells in females than males in the AVPV (anterior ventral periventricular area) (Fig. 1B). Using dual-immunoflourescence studies, we further showed that some PKR2-expressing neurons appeared to coexpress estrogen receptor (ERα) (Fig. 5). While explicit cellular colocalization of PKR2 and Erα has not been resolved, they are at least expressed in the AVPV (Fig. 5). The sexually dimorphic expression of PKR2 in the preoptic area and the likely co-localization with ERα suggests that PK2/PKR2 signaling may play a signaling role in the reproductive axis.

We, and others, have developed mutant mice that are deficient in PK2 or PKR2 genes [12], [18]. As the developmental defect of GnRH neurons in PK2-null as well as PKR2-null mice precludes the use of such null mutant mice for the analysis of a signaling role at adult-stage physiology, we carefully examined the estrous cycle of mutant mice with loss of a single copy of PK2 or PKR2 (Fig.3). Compared to wild-type, littermate controls, PK2+/− mice displayed slightly increased estrous cycle length (5.4±1.9 days), compared to controls (4.5±0.5 days). PKR2+/− mice exhibited significantly more abnormal estrous cycles, with the mean estrous cycle length (7.7±3.5 days, P<0.05). The PK2+/−, PKR2+/− double heterozygous mice showed the most severe phenotype, with all female mice exhibiting anomalous cycles (either longer, or irregular cycles or completely non-cycling). Average estrous cycle length in these double-heterozygous mice (8.8±4.8 days) was nearly twice as long as that of wild type females (p<0.01). In particular, we observed elongated diestrous stages in double-heterozygous mice, and four out of six females were essentially non-cycling. The increased severity in double heterozygous mice, compared to that of single PK2 or PKR2 heterozygous mice, is consistent with the interaction of PK2 with PKR2. Previous studies have revealed about 50% reduction in the expression levels of PK2 or PKR2 in heterozygous mice [18]. The coincident reduction of expression of both PK2 and PKR2 genes would then result in aggregate defect in PK2-PKR2 signaling, and thus more pronounced deficit in the estrous cycles.

**Figure 6:**
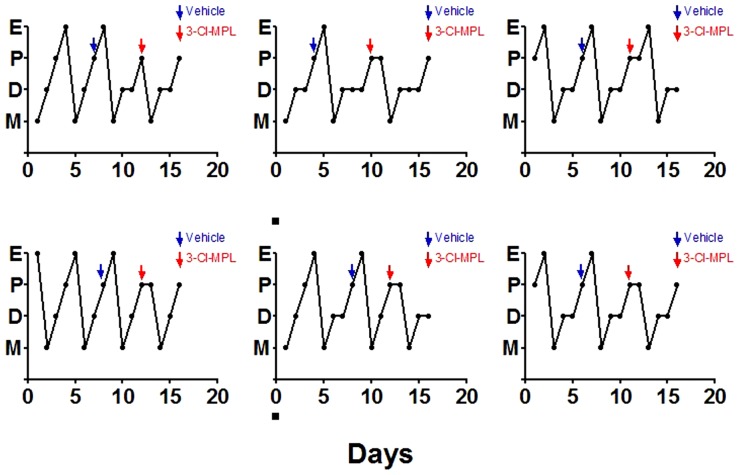
Potency of PKR2 antagonist, 3Cl-MPL, in antagonizing PKR2. Antagonist potency was examined in Chinese Hamster Ovary (CHO) cells that stably express PKR2. RLU is an index for calcium influx measurement with a luminescence-based assay. The IC50 of 3Cl-MPL for PKR2 were 24.9±4.3 nM(Mean ± S.E.). Shown was representative of three independent experiments.

**Figure 7:**
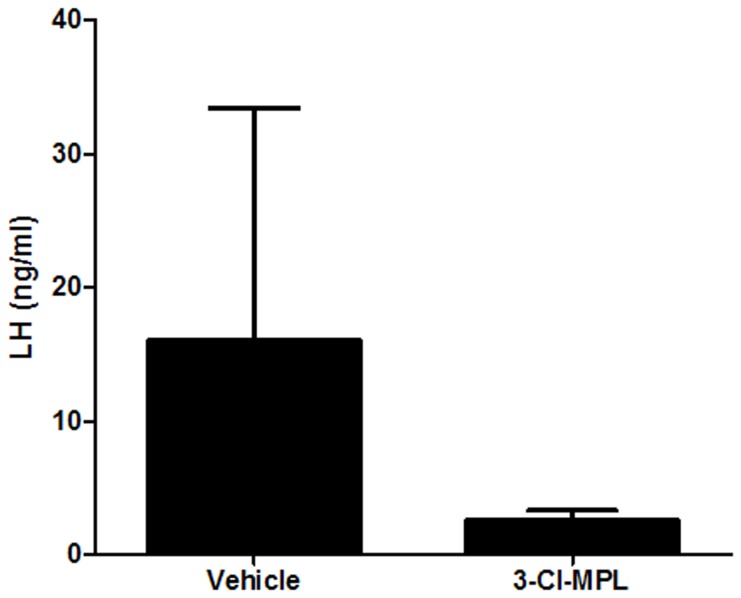
Blocking of estrous cycle by PKR2 antagonist treatment. The administration of 3Cl-MPL (shown in red arrows) prevented the progression to estrous stage. Vehicle treatment was shown by blue arrows. E: estrus, P: proestrus, D: diestrus, M: metestrus.

To rule out the possible involvement of any developmental defects of GnRH neurons, we documented that cell counts of GnRH neurons within the hypothalamus in WT, PK2, PKR2 single- and compound-heterozygous female mice. As shown in Fig. 4, cell counts of GnRH neurons in these single- and compound-heterozygous female mice were not significantly different, indicating that the abnormality of estrous cycling was not due to any gross developmental defect. Recently, we have developed a series of PKR2 antagonists [29], [30], which permits blockade of PKR2 signaling without any confounding effects on development. These antagonists inhibit the signaling of PK2-stimulated calcium mobilization in Chinese Hamster Ovary (CHO) cells that stably express PKR2 with high potency. One such PKR2 antagonist, 3Cl-MPL, with IC50 of 24.9±4.3 nM for PKR2, was used for the current study (Fig. 6). We tested the effect of 3Cl-MPL on the function of female reproductive cycle by administering a single dose of PKR2 antagonist in the morning of the proestrous stage to wild type C57BL6 mice. Figure 7 shows that, in contrast to the vehicle control, PKR2 antagonist led to the temporary blockade of estrous cycle in essentially all animals. Particularly, the proestrous phase was elongated at the cost of the estrous phase (Figure 7). This effect is in line with the alterations observed in the estrous cycles of PK2 or PKR2 single- and compound-heterozygous female mice. We further examined the effect of PKR2 blockade on the LH levels by administering PKR2 antagonist. As shown in Figure 2, the LH levels at ∼ZT12 in the control group were 8.91±3.83 ng/ml, while the LH level in the group receiving antagonist were significantly reduced (1.09±0.74 ng/ml. P<0.01). This result indicates that administration of the PKR2 antagonist reduced the circulating levels of LH at the time of lights off, suggesting either a reduction in the amplitude or a delay in the timing of the LH surge by PKR2 blockade.

## Discussion

To maintain a normal estrous cycle is important for the fertility of females. The pre-ovulatory GnRH surge is primarily controlled by two types of inputs to GnRH neurons: hormonal feedback from maturing ovarian follicles and circadian outputs from the SCN [2], [31], [32]. On the afternoon of proestrus, a GnRH surge release from the hypothalamus induces the pituitary to release LH and FSH, which act on the ovary to induce ovulation and follicular recruitment. SCN lesion studies and estrous cycle defects in the Bmal1 null mice supported the importance of circadian output from SCN for the normal expression of the estrous cycle[4]. Several signaling molecules, especially vasopressin and vasoactive intestinal polypeptide, have been implicated as SCN output signals that link to the reproductive axis [31], [32], [33], [34].

The current studies investigated the effect of PK2, also established as a SCN circadian clock output molecule [10], [17], [18], [35], in the normal expression of the estrous cycle in female mice. The sexually dimorphic expression pattern of PKR2 and coexpression of PKR2 with ERα in the preoptic area suggest that PK2-PKR2 signaling in the brain may likely be involved in the regulation of the reprodutive cycle. We have obtained genetic and pharmacological evidence to support this hypothesis. Loss of one copy of PK2 and/or PKR2 genes caused elongated and irregular estrous cycles in female mice, with the abnormality even more pronounced in compound-heterozygous mice. Consistent with this observation, pharmacological blockade of the PK2-PKR2 signaling with a PKR2 antagonist led to the blunted circulating LH levels and temporary blocking of the estrous cycle in the majority of animals. Taken together, these observations indicate PK2 signaling is needed for the normal expression of the estrous cycle in female mice. A lower level of cycling still existed in some compound-heterozygous females and antagonist-treated mice, which is consistent with the notion that circadian signaling is facilitatory, but not absolutely required for estrous cycles, i.e. in the absence of circadian output signals, female cycling can occur in some degree. While our results indicate the likely signaling role of PK2 is due to the effect of PK2 as a SCN circadian clock output molecule, PK2 is indeed expressed in several other brain areas, and we could not rule out the possibility this effect of PK2 on the regulation of female estrous cycle is due to non-SCN PK2.

The molecular mechanism of SCN output is an area of active investigation. In addition to PK2, at least two other clock-controlled regulatory peptides (vasopressin, and cardiotrophin) may function as signaling molecules that convey SCN timing information [9], [10], [11]. Vasopressin is expressed in neurons projecting from the SCN to the preoptic area [7], [8], [9], and inhibition of vasopressin signaling on the morning of the proestrous stage significantly attenuates LH release [8]. Further genetic studies also indicated that reduced hypothalamic vasopressin signaling plays a role in the proestrous LH surge [36]. The majority of SCN neurons appears to use GABA as a neurotransmitter[37], and the release of GABA during proestrus exhibits a circadian pattern [38]. Though it is unclear how these clock-controlled genes interact with each other or with GABA, redundant circadian signals could regulate the GnRH surge, underscoring the significance of a finely tuned reproductive cycle that is adaptive to environmental cues.

GnRH neurons express the receptor for Kisspeptin (GPR54) but not PKR2 [15], [39], [40]. Several studies have revealed that up-regulation of Kisspeptins by estrogen in neurons of the AVPV region preceeds the GnRH release that stimulates the LH surge required for ovulation [25], [26], [41], [42]. Kisspeptin could stimulate GnRH release directly by binding to its receptor on GnRH neurons [39], [43], [44]. The sexually dimorphic expression pattern of PKR2 in the preoptic area is, however, very similar to that of Kisspeptin which is known to play essential roles in stimulating the pulsatile release of GnRH at puberty[25], [26], [27]. Moreover, some neurons in the AVPV seem to coexpress PKR2 with ERα. Similar co-expression pattern with estrogen receptor (ERα) was also observed for the kisspeptin-expressing neurons[28]. As it has been shown that the activity of Kisspeptin neurons in the AVPV exhibit a circadian-controlled activity [33], [45], it is thus intriguing to examine whether PK2-PKR2 signaling could transmit the circadian clock information to Kisspeptin-neurons in the AVPV region for reproductive control.
