# Correction: Signaling Role of Prokineticin 2 on the Estrous Cycle of Female Mice

**DOI:** 10.1371/journal.pone.0098314

**Published:** 2014-05-15

**Authors:** The PLOS ONE Staff

Figures 2, 5, 6, and 7 are in the incorrect order. The publisher apologizes for these errors. The figure legends are correct. The correct versions of Figures 2, 5, 6, and 7 can be viewed here.

**Figure 2:**
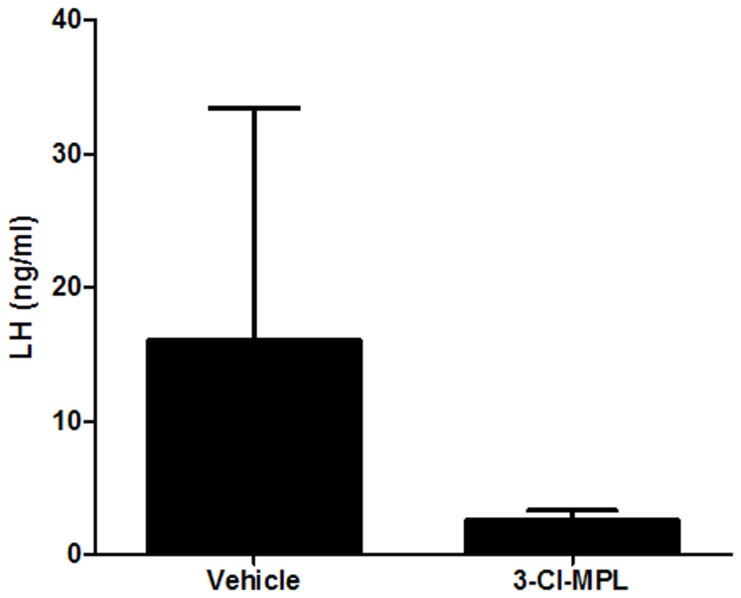
PKR2 antagonist reduced plasma LH levels (Mean ± S.E.). The LH levels in the PKR2 antagonist (10 mg/kg 3Cl-MPL) group were significantly reduced compared to the vehicle treatment (n  =  6 for each group, p<0.01.

**Figure 5:**
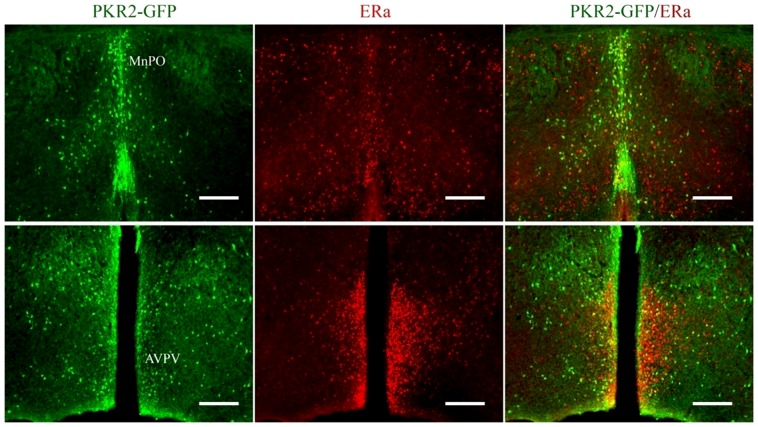
The coexpression of PKR2 and ERα in the preoptic area. PKR2 and ERα were detected by immunofluorescence staining. PKR2-GFP expression was shown in green and ERα expression was shown in red. Yellow or orange color in the MnPO and AVPV regions indicates likely co-expression of PKR2 and ERα (scale bars:100 µm).

**Figure 6:**
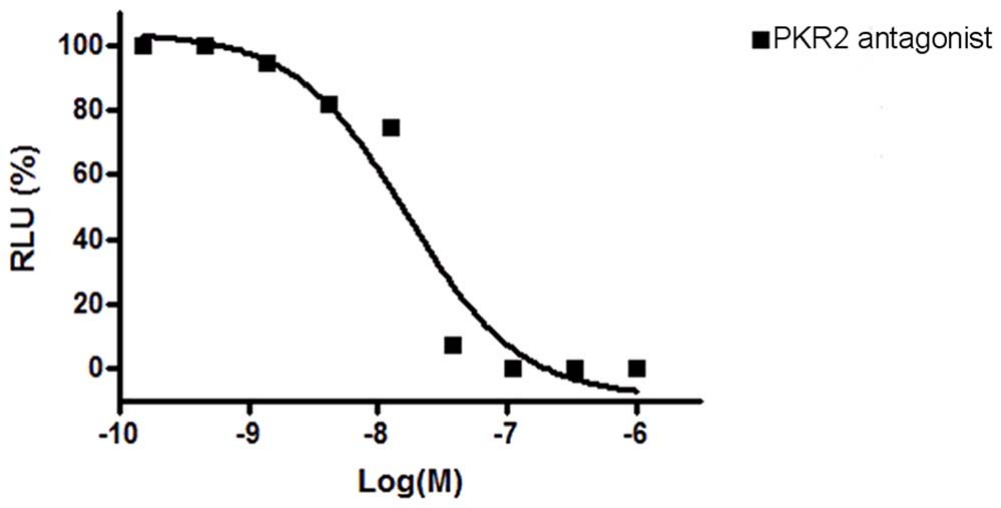
Potency of PKR2 antagonist, 3Cl-MPL, in antagonizing PKR2. Antagonist potency was examined in Chinese Hamster Ovary (CHO) cells that stably express PKR2. RLU is an index for calcium influx measurement with a luminescence-based assay. The IC50 of 3Cl-MPL for PKR2 were 24.9±4.3 nM(Mean ± S.E.). Shown was representative of three independent experiments.

**Figure 7:**
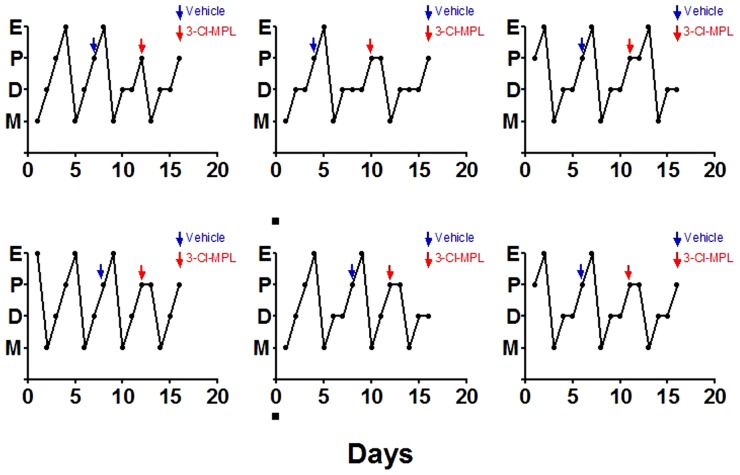
Blocking of estrous cycle by PKR2 antagonist treatment. The administration of 3Cl-MPL (shown in red arrows) prevented the progression to estrous stage. Vehicle treatment was shown by blue arrows. E: estrus, P: proestrus, D: diestrus, M: metestrus.
